# Supplementary material for: Structures of insect Imp-L2 suggest an alternative strategy for regulating the bioavailability of insulin-like hormones
Source: Nat Commun. 2018 Sep 21;9:3860. doi: 10.1038/s41467-018-06192-3 (PMC6155051; doi:10.1038/s41467-018-06192-3)
Supplement: Supplementary file 1 — Supplementary Information [file 41467_2018_6192_MOESM1_ESM.pdf]

Supplementary Information for:

Structures of insect Imp-L2 suggest an alternative strategy for regulating the bio-availability of insulin-like hormones

Roed et al.

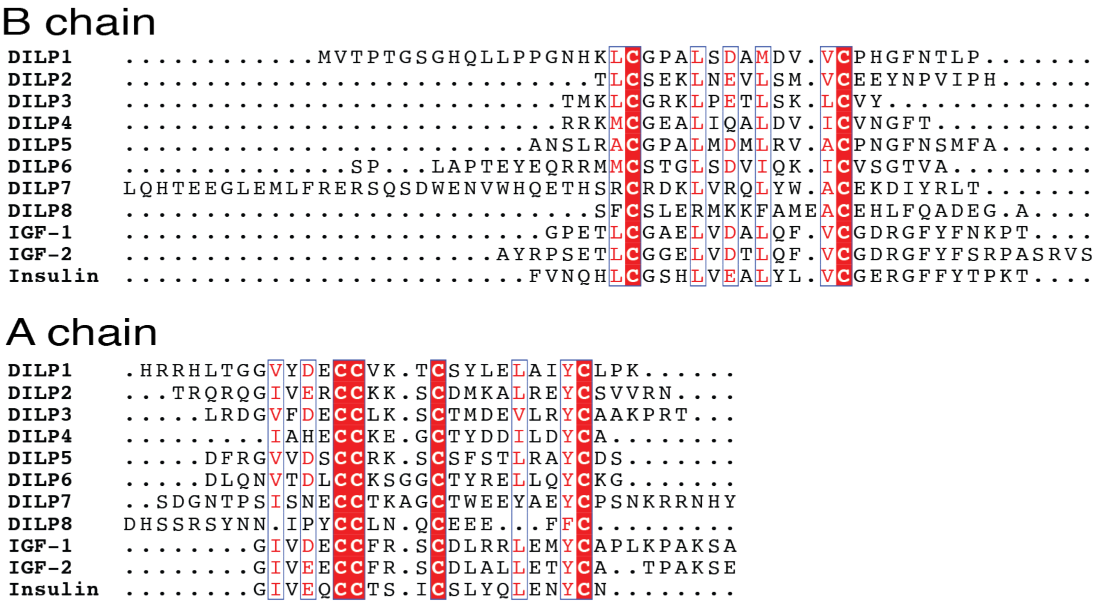

Supplementary Figure **1. Sequence alignment of the A and B chains of human insulin, IGF-1, IGF-2, and all DILPs (1-8) from *Drosophila melanogaster*.**

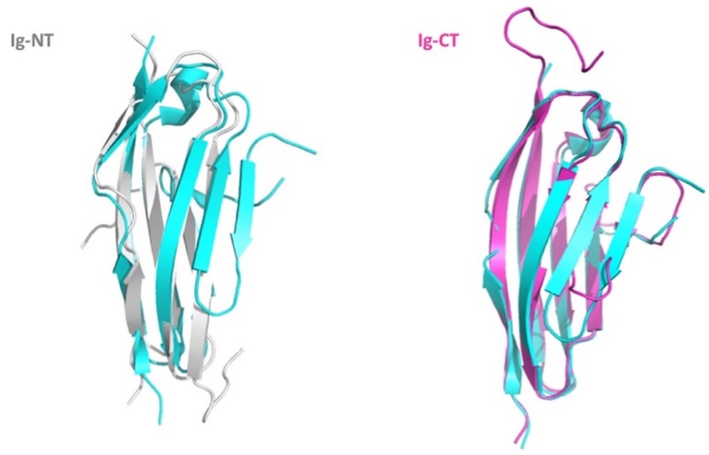

Supplementary Figure **2. Comparison of the fold of the Ig-NT and Ig-CT domains of the Imp-L2 ((in grey and in magenta, respectively) with M10$^{1-99}$ domain of human titin (PDB ID 3q4o, in blue).** The structures have been superposed by the Global SSM superposition option in Coot [1].

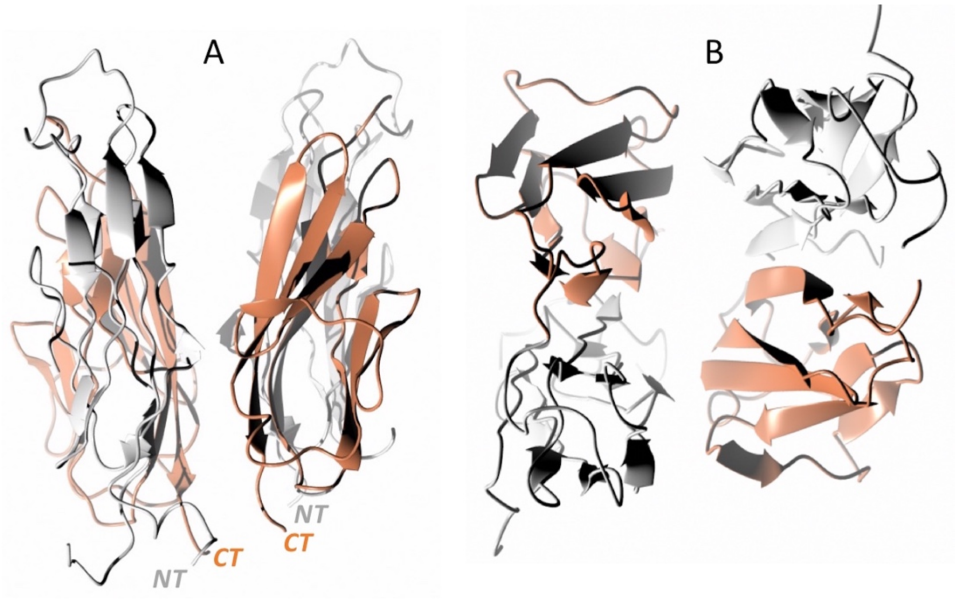

Supplementary Figure **3. Top-to-top arrangement of the apo-Imp-L2 dimer.** A. The edge view of the dimer; B. the top view after rotation of dimer in A by 90° along horizontal axis. The NT-domains are in white and CT-domains in coral, NT/CT: termini of the protein.

Supplementary Table **1. Summary of the main hormone:Imp-L2 interactions.** The interacting groups of the side/main chains are given next to the name/number of the amino acid: DILP5 – first column, Imp-L2 second column; sc – side chain, mc – main chain, VDW – van der Waals interactions (weak due to ~3.6 – 4 Å distances between groups that interact, or may be involved in potential VDW contact with better definition of the side chains and small protein movement), HB – hydrogen bond.

Hormone	Imp-L2	Type of interaction
**DILP5**		
LeuB10 sc	Tyr235 sc	weak VDW
LeuB10 sc	CysC6 sc	weak VDW
MetB13 sc	Leu159 sc	weak VDW
MetB13 sc	Ile93 sc	weak VDW
AlaB17 mc	Met38 sc	weak VDW
AlaB17 mc	Trp32 sc	weak VDW
CysA9 CO	Tyr235 OH	HB
ArgA11 CO	Trp211 NE1	HB
SerA13 CO	Leu238 NH	HB
PheA16 sc	Leu159 sc	weak VDW
PheA16 sc	Ile93 sc	weak VDW
PheA16 sc	Val237 sc	weak VDW
PheA16 sc	Ile161 sc	weak VDW
LeuA19 sc	Leu159 sc	weak VDW
**IGF-1**		
Glu9 OE1	Asn216 ND2	HB
Phe16 CO	Lys34 NZ	HB
Ser51 OG1	Pro236 CO	HB
Lys52 CO	Leu238 NH	HB
Gly1 mc	Met214 sc	weak VDW
Pro2 mc	Met214 sc	weak VDW
Cys6 sc	Phe233 sc	weak VDW
Cys6 sc	Met214 sc	weak VDW
Leu10 sc	Phe233 sc	weak VDW
Ala13 sc	Met58 sc	weak VDW
Val17 sc	Trp32 sc	weak VDW
Val17 sc	Met58 sc	weak VDW
Arg50 mc	Trp211 sc	weak VDW
Ser51 mc	Trp211 sc	weak VDW
Leu57 sc	Tyr235 sc	weak VDW

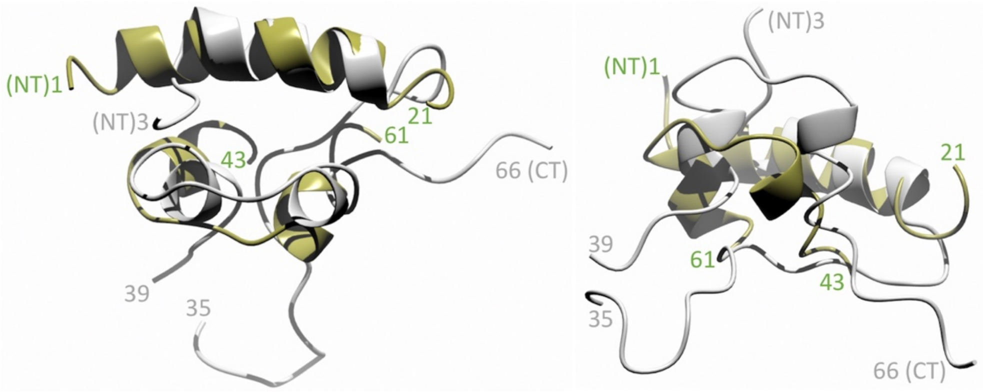

Supplementary Figure **4. Comparison of the free IGF-1 (PDB ID 1gzr - white) and Imp-L2 bound IGF-1 structures (green)**. The NT/CT indicate the respective termini of the hormones. The R-like conformation of the IGF-1 in the Imp-L2 complex is especially clear in the projection in the left panel, when the large shift of the IGF-1 A-helices (43-61) upon binding to Imp-L2 is clearer on the right panel. Superposition has been done on the 10-18 segments of the B-helices (Cα atoms) by the LSQ option in Coot [1].

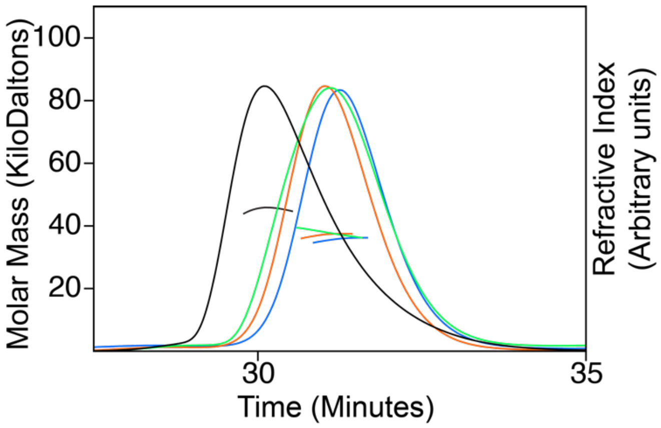

Supplementary Figure **5. Molecular masses of three hormone mixtures with IMP-L2 measured from SEC-MALLS analysis.** 100 µL of 1 mg• mL^{-1} protein mixtures were loaded onto a Superdex S200 10/300 gel filtration column. An overlay of four different traces of refractive index as a function of time are represented by the following curves: apo Imp-L2 (black), DILP5 (green), IGF-1 (red) and insulin X14 (blue).

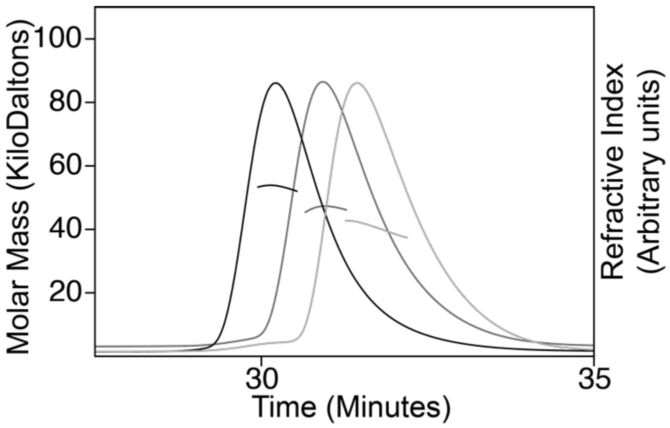

Supplementary Figure **6. Molecular masses measured from SEC-MALLS analysis at three different salt concentrations.** 100 µL of 1 mg• mL^{-1} protein samples incubated in three different concentrations of NaCl were loaded onto a Superdex S200 10/300 gel filtration column. An overlay of the three different traces of refractive index as a function of time in 50 mM, 150 mM and 400 mM NaCl (left to right) are represented by black, dark grey and light grey respectively.

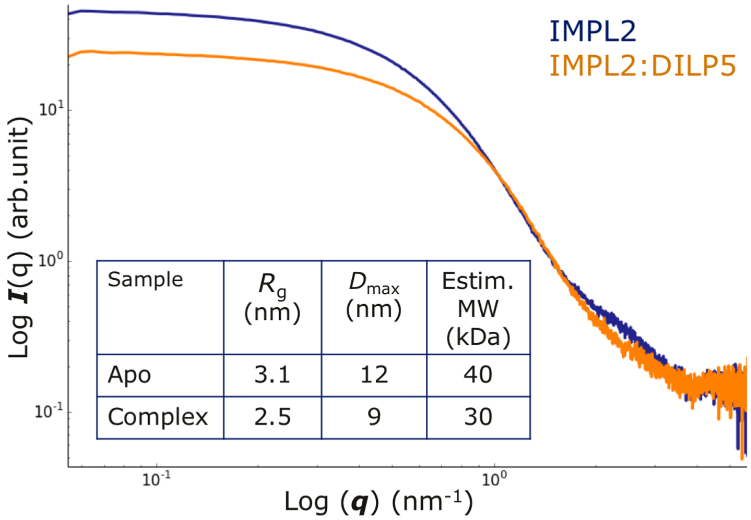

Sample	R_g (nm)	D_{max} (nm)	Estim. MW (kDa)
Apo	3.1	12	40
Complex	2.5	9	30

Supplementary Figure **7. Small Angle X-ray scattering curves of Imp-L2 (blue) and Imp-L2:DILP 5 complex (orange).** Plotted is log of the intensity ***I*** vs. the log of the scattering vector ***q***. The insert table shows radius of gyration (R_g), maximum distance (D_{max}), and the estimated molecular weight of the protein, as derived from the scattering curves by ATSAS [2].

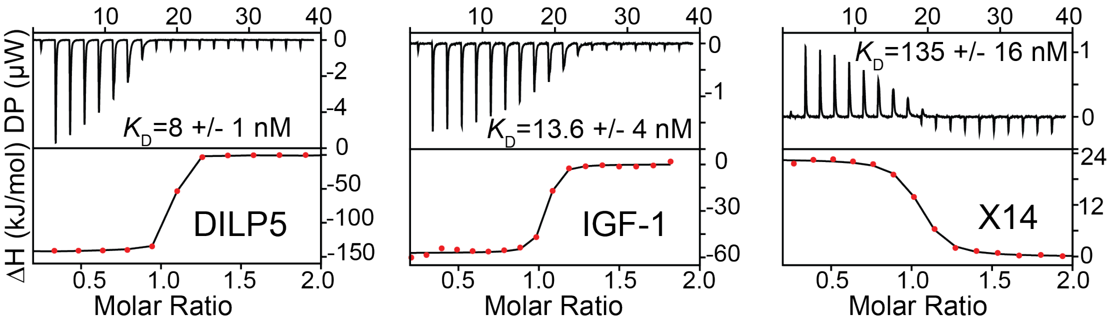

Supplementary Figure **8. Typical ITC traces for the interaction between Imp-L2 and DILP5, human IGF-1 and insulin X14.** Top panels are titration data obtained from nineteen 2 µL injections of hormones (DILP5: 180 µM, IGF-1: 119 µM, insulin X14: 269 µM) into Imp-L2 (12.8 µM, 13 µM and 22.6 µM, respectively). All injections were carried out in 50 mM Tris, 150 mM NaCl, pH 7.4 buffer. Bottom panels are integrated heats (red circles) and the heat expected from the fitted One Site model. Error bars (Supplementary Figure **9**) and +/- s.d. were calculated by MicroCal PEAQ-ITC Analysis software (Malvern).

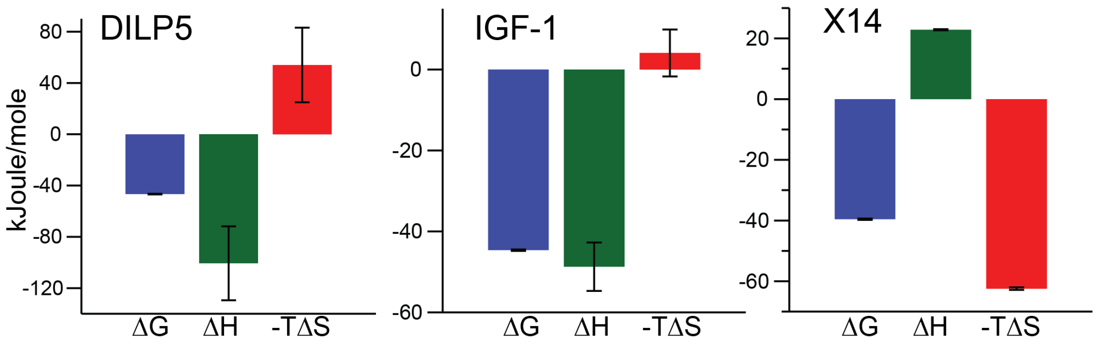

Supplementary Figure **9. Thermodynamic signatures of DILP5, IGF-1 and insulin X14 interactions with Imp-L2 derived from ITC measurements presented in Supplementary Figure 8.**

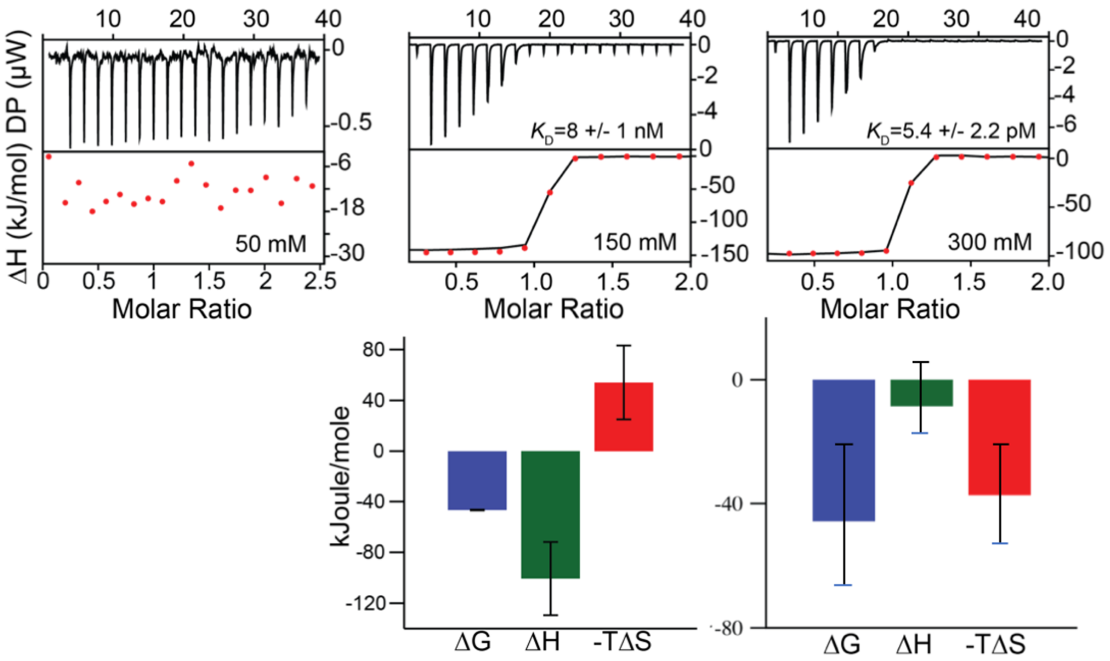

Supplementary Figure **10. DILP5:Imp-L2 binding as a function of ionic strength.** Top – ITC measurements, bottom – corresponding thermodynamic profiles. No DILP5 binding is observed at 50 mM NaCl, while 400 mM NaCl environment shits DILP5 affinity into a pM range despite a large experimental error, typical for the ITC method at such high affinity.

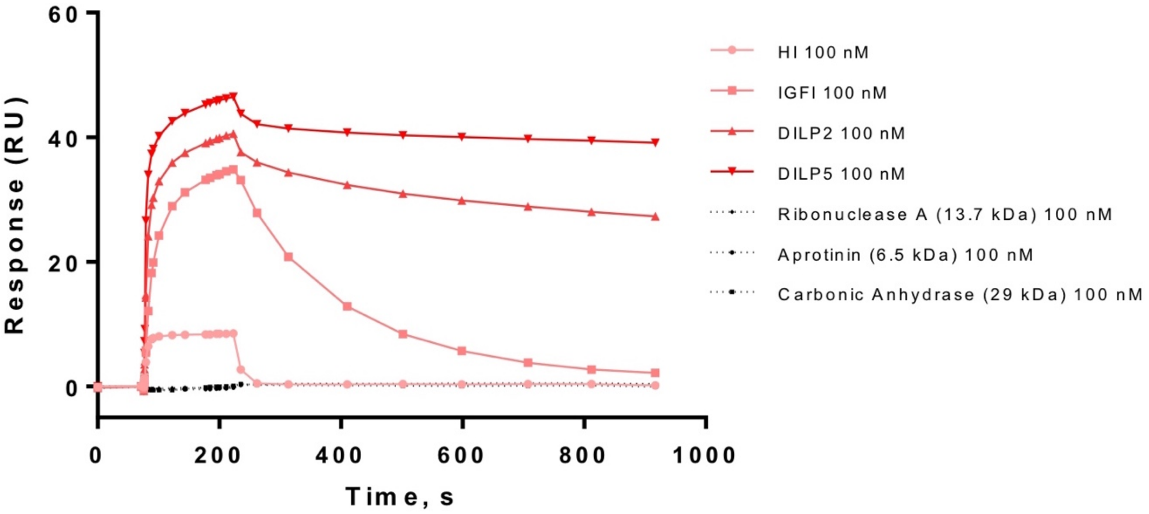

Supplementary Figure **11. SPR analysis of binding of immobilised Imp-L2, to human insulin and IGF-1, DILP2 and DILP5.**

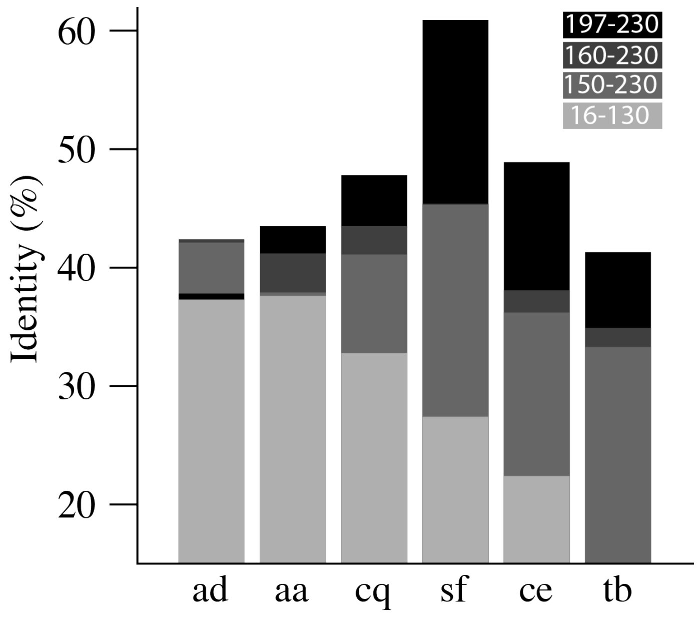

Supplementary Figure **12. Sequence identity between the segments of Imp-L2 defined in the top right corner and IBPs listed in main text Figure 7.** ad – *Anopheles darlingi*, aa – *Aedes aegypti*, cq – *Culex quinquefasciatus*, sf - *Spodoptera frugiperda*, ce – *Caenorhabditis elegans*, tb - *Trichinella britori*. Top right corner insert: Imp-L2 sequence segments (and their colour coding used in this graph) selected for the alignments with the corresponding sequences of other IBPs listed here.

Supplementary Table **2. Two sets of primers used for cloning and production of the Imp-L2.** The insert set of primers was used for amplifying the Imp-L2, and the backbone set of primers was used for amplifying the pBac4x vector. The uppercase-letters are the bases that are complementary to the opposite strand.

insert forward	5'-AAAACCGCCTGGATGatttacatgtgtgcgccttagcg
insert reverse	5'-GGAGAAGGCGCGTTActtttcgaactgggggtgg
backbone reverse	5'-CATCCAGGCGGTTTTttaggagttc
backbone forward	5'-TAACGCGCCTTCTCCtcagg

Supplementary Table **3. X-ray data collection and refinement statistics.**

	Imp-L2	Imp-L2:DILP5	Imp-L2:IGF-1
**PDB Code**	4CBP	6FEY	6FF3
**Data collection**			
Beamline	MAXII, I911-3	DLS, i24	DLS, i03
Wavelength (Å)	0.9800	0.91500	0.97625
Space group	$P2_1$	$P4_12_12$	$C2$
Cell dimensions			
a, *b*, *c* (Å)	49.86 99.73 56.28	150.83 150.83 125.32	82.03 94.15 48.36
α, β, γ (°)	90.0 92.0 90.0	90.0 90.0 90.0	90.0 95.3 90.0
Resolution (Å)	56.3-1.59(1.65-1.59)	29.6-3.48(3.67-3.48)	48.16-2.57(2.64-2.57)
R_{sym}	0.05(0.42)	0.165(1.17)	0.093(0.783)
R_{pim}	-/-	0.089(0.63)	0.059(0.47)
$<I / \sigma(I)>$	13.4(2.3)	8.0(1.4)	7.6(1.0)
Completeness (%)	95.7(71.9)	98.9(94.3)	96.4(97.6)
Redundancy	3.5(2.3)	4.3(4.3)	3.9(4.1)
**Refinement**			
Resolution (Å)	29.54-1.60	42.89-2.02	37.34 - 2.15
No. of reflections	69600	17857	10666
R_{work} / R_{free}	0.157/0.188	0.261/0.345	0.232/0.285
No. atoms	4340	6187	1900
Protein	3869	5619	1886
Ligand	14	-	-
Ions	-	-	-
Water	457	4	14
B-factors			
Protein	26.4	103.3	61.7
Ligand	41.4	-	-
Ions	-	-	-
Water	38.6	42.0	58.9
R.m.s. deviations			
Bond lengths (Å)	0.006	0.011	0.013
Bond angles (°)	1.086	1.734	1.676
**Ramachandran (%)**			
Preferred/Allowed/ Outliers	95.7/3.5/0.8	90.4/6.8/2.8	95.4/4.2/0.4

*Values in parentheses are for highest-resolution shell.

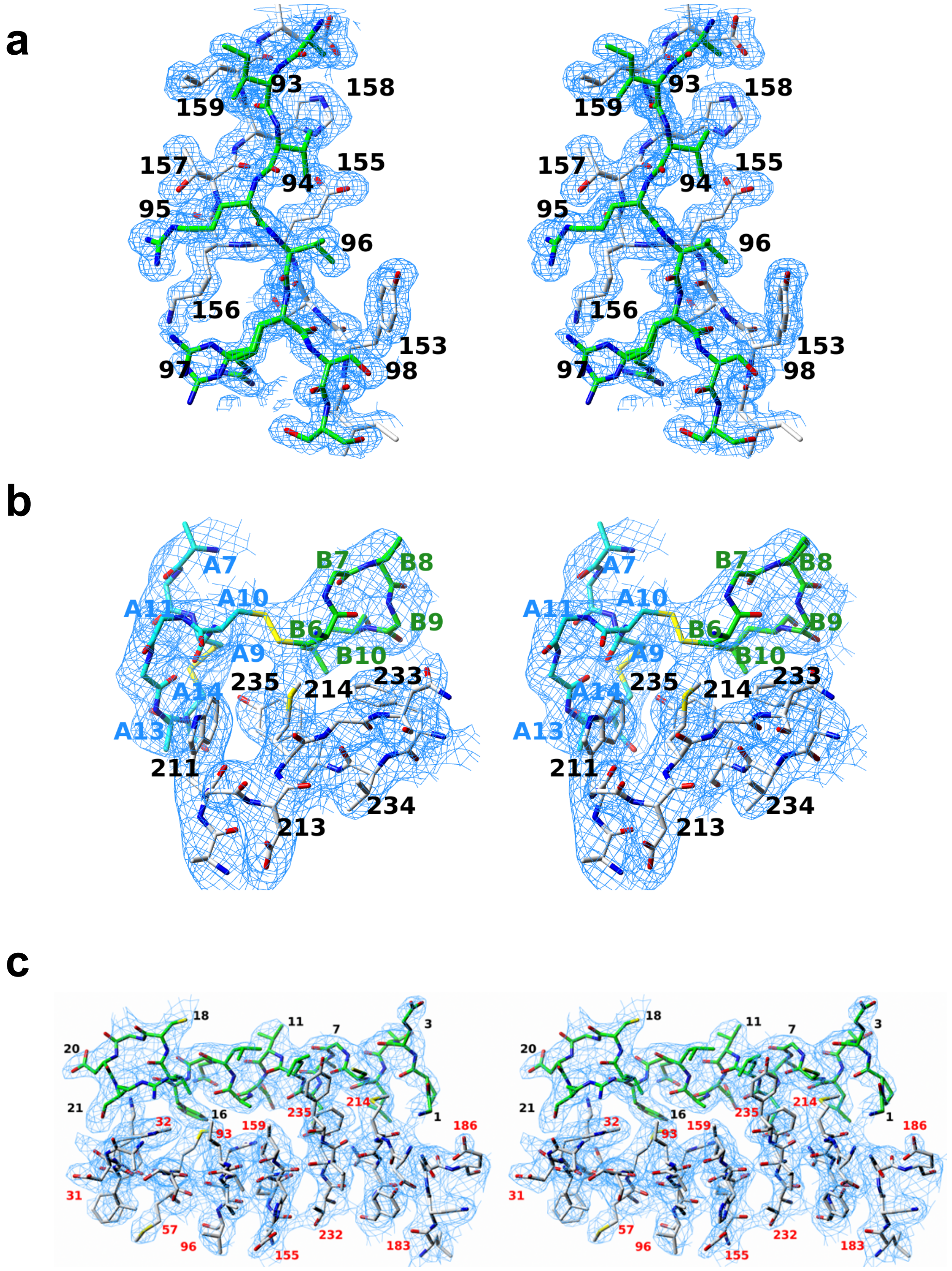

Supplementary Figure **13. Stereo representation of typical Refmac weighted 2m*F*o-D*F*c electron density maps for crystal structures presented in this work. a** apo-Imp-L2, **b** DILP5:Imp-L2 complex, and **c** IGF-1:Imp-L2 complex. All maps contoured at 1σ level. In **a** the 93-98 and 153-159 part of the id-β-sheet interface is shown; the Cα atoms of the 93-98 and 153-159 strands are in green and white, respectively. In **b** some important DILP5-Imp-L2 contacts (especially the Imp-L2 Met214 - hormone B6-A10 disulphide) contacts are shown; Cα atoms of the B-chain (green labels), A-chain (blue labels) and Imp-L2 (black labels) are in green, blue and white, respectively. Panel **c** depicts arrangement of IGF-1 B-helix segment (Cα atoms in green, black labels) with part of the Imp-L2 id-β-sheet (Cα atoms in white, red labels).

Supplementary References.

1. Emsley, P. & Cowtan, K. Coot: model-building tools for molecular graphics. *Acta Crystallogr. D Biol. Crystallogr.* **60**, 2126-32 (2004).

2. P. V. Konarev, M.V.P., V. V. Volkov and D. I. Svergun. ATSAS 2.1, a program package for small-angle scattering data analysis. *J. Appl. Cryst.* **39**, 277-286 (2006).
